# A Review of *Diopatra* Ecology: Current Knowledge, Open Questions, and Future Threats for an Ecosystem Engineering Polychaete

**DOI:** 10.3390/biology11101485

**Published:** 2022-10-11

**Authors:** Sarah K. Berke

**Affiliations:** Siena College, Department of Biological Sciences, Loudonville, NY 12211, USA; sberke@siena.edu

**Keywords:** facilitation, ecosystem engineering, Annelida, human impacts, marine sediments, infauna, community ecology

## Abstract

**Simple Summary:**

Most of the world is ocean, and most of the ocean bottom is mud or sand. Understanding the ecology of sedimentary habitats is therefore important for understanding marine ecosystems writ large. Marine sediments are typically occupied by burrowing and tube-building animals that physically structure the habitat. In coastal sediments, an especially widespread example is the annelid worm *Diopatra*, which builds large tubes up to 2 m deep and 1 cm in diameter. These tubes have extensive physical effects on other organisms in the habitat, including commercially important fish and crustaceans. *Diopatra* are currently being impacted by climate change, species invasions, and (in some areas) the bait-digging industry. In this article, I review what we know about *Diopatra* ecology with an eye to identifying major open questions and future threats facing this important architect of coastal marine systems.

**Abstract:**

A well-known example of marine ecosystem engineering is the annelid genus *Diopatra*, which builds large tubes in coastal sediments worldwide. Early studies of *Diopatra* were among the first to recognize the importance of facilitation in ecology, and *Diopatra* has become a key marine soft-sediment application of the ecosystem engineering concept. Here, I review our current knowledge of *Diopatra* ecology, including its natural history, ecosystem engineering effects, and trophic relationships. I particularly explore how human activities are influencing *Diopatra* in terms of climate change, bait fishing, and species invasions. Most of what we know about *Diopatra* ecology comes from focal studies of a few species in a few well-known regions. Further evaluating how our current understanding applies to other species and/or other regions will help to refine and deepen our understanding of structure and function in marine systems.

## 1. Introduction

**Figure 1:**
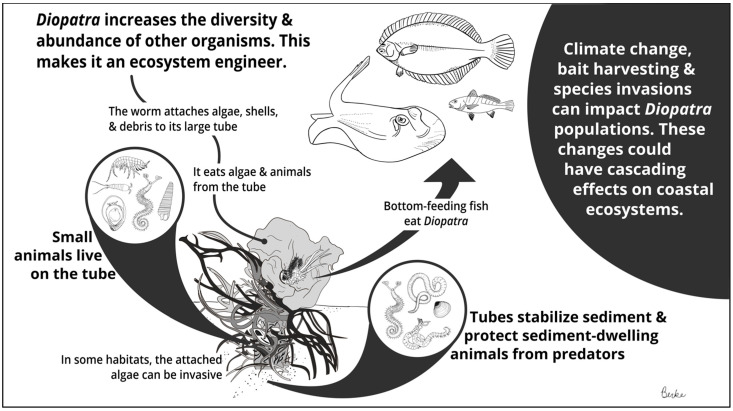
Summary of *Diopatra* ecological interactions.

Onuphid polychaetes in the genus *Diopatra* have captured scientific interest for more than two centuries. The earliest descriptions of *Diopatra* [1,2,3] remarked on the worm’s size, beauty, and charismatic habit of festooning its tube with fragments of shell, drift algae, and other debris; by the mid-20th century, ecologists increasingly recognized that *Diopatra* is not only charming but also ecologically important. Some of the earliest examples of facilitation between species were reported for *Diopatra* [4]. Such effects, now regarded as ecosystem engineering [5,6], are largely due to the physical and biological impacts of the worm’s tube structure (Figure 1). *Diopatra’s* robust tubes descend deep into the sediment, emerging above the surface in a decorated ‘tube cap’ [7,8]. *Diopatra* tube caps provide physical structure in habitats where structure is scarce, thereby facilitating a wide variety of epibenthic organisms [9,10,11]. The tubes also stabilize sediments and create predation refugia for infauna [4,12]. Here, I review our current knowledge of *Diopatra* ecology, including its natural history, role as an ecosystem engineer, and responses to anthropogenic change. My goals are to synthesize existing knowledge and to identify open questions regarding this interesting genus. 

### Taxonomic Caveat

Polychaete taxonomy in general—and *Diopatra* taxonomy specifically—is in a period of especially rapid revision [13,14,15,16]. Like many polychaetes, the earliest-recognized *Diopatra* species were historically thought to show cosmopolitan distributions. The name *D. neapolitana* has been applied to worms from Europe, the Mediterranean, Africa, and Southeast Asia [8,17,18,19]. Similarly, the name *D. cuprea* has been applied to worms throughout North America, Central America, Brazil [11,20], the eastern Atlantic [21], and the Indian Ocean [22,23]. We now recognize that such cosmopolitan distributions are quite unlikely [14]. Old species descriptions are being rapidly split and revised as modern systematists, armed with molecular toolkits, generate a cornucopia of new species. This work is revealing enormous diversity within the *Diopatra* genus [13,15,16,24,25], and, undoubtedly, more species await description.

Many important contributions to *Diopatra* ecology were made in publications that pre-date this recent burst of novel taxonomic insight, raising questions about which species were actually the focus of any given study. Although this is a real challenge, we should not lose sight of the enormous value provided by these ecological studies. In this review, I will use the species name associated with the original publication. In cases where the original name is clearly not correct (i.e., uses of *D. neapolitana* for regions far from western Europe and the Mediterranean), I will indicate this by simply using the genus name and including a brief remark about the named used in the study. Anyone working on *Diopatra* should stay abreast of taxonomic developments in their geographic region. Ecologists would do well to keep voucher specimens and tissue samples for genetic analysis whenever possible. Increased collaboration between ecologists and taxonomists would be most welcome.

## 2. Natural History

*Diopatra* species occur in temperate and tropical coastal and estuarine systems worldwide [18,25,26,27,28,29]. Like most estuarine species, *Diopatra* exhibits wide salinity tolerance from 15 to 40 ppt, and at least some species can survive short periods outside that range [30]. Typical habitats include protected intertidal and shallow subtidal flats of muddy sand or sandy mud—generally not pure sand, pluff mud, or clay [1,31]. Within a geographic region, *D. cuprea* density is higher in areas with faster currents, but this trend breaks down when drawing comparisons across geographic regions [31]. The best-known *Diopatra* species occupy a tidal range from the mid intertidal to the shallow subtidal [11,32], but some species occur at greater depths, e.g., *D. ornata* in the eastern Pacific, *D. leuckarti* in Hawaii [29], and multiple new species recently described from the continental shelf of Africa [16].

*Diopatra* is notable for constructing, in Verrill’s words, “a very curious permanent tube in which it dwells very securely” [1]. Most of the worm’s ecosystem engineering effects are tied to this tube-building behavior. The tube extends downward 1 m or more beneath the sediment. The above-sediment portion of the tube varies across species but typically emerges several centimeters above the sediment surface and is decorated with shell fragments, detritus, and algae [13], with the aperture opening either perpendicular to the sediment or pointing down at the sediment [33,34,35]. Exceptions to this typical phenotype include *D. budaevae* and *D. hektoeni,* both of which exhibit thinner, more fragile (but still decorated) tubes [13], and *D. neapolitana*, which constructs tubes either flush with the sediment surface or only slightly emergent, with little to no decoration [13,36]. Whereas most *Diopatra* occur singly, at least two species, *D. leuckarti* and *D. ornata*, have been described as forming mounds or reefs of elevated sediment [29,37]. Tube morphology is a useful field characteristic; whenever possible, descriptions and photographs of tubes should be included in taxonomic descriptions. Tube morphology can also vary within a species; data about the range of decoration and tube size for a given species (as collected by Wethey et al. in their 2016 study [36]) are helpful, particularly if they can help disambiguate co-occurring species in the field.

New recruits build a tube upon settlement, and the tube is expanded (both in diameter and depth) as the animal grows. The tube’s innermost layers consist of a parchment-like material formed by a mucous secretion that polymerizes in seawater. The outermost layer of the tube consists of foreign matter imbricately cemented to the structure, with each object extending outward from the tube wall. To attach an item, the worm holds it in position with its jaws, palps, and anterior-most parapodia (softer materials are sometimes first trimmed with the jaws), then glues it into place by rubbing its anterior ventral surface over the attachment point, secreting mucous from glands beginning at the sixth segment [2]. Myers [34] found that *D. cuprea* prefers ‘tabular’ materials and appears to utilize progressively smaller items along the axis from the tube opening toward the sediment surface, otherwise selecting material indiscriminately. Preferences have not been assessed for other species.

Why does *Diopatra* decorate its tube? Algae and invertebrates found on *D. cuprea* tubes are also found in its gut, suggesting that ‘gardening’ plays a major role (reviewed in [11,38]). Decoration extending from the tube cap may function like ‘whiskers’ to help the worm detect disturbance and avoid predation [39]. Some data for *D. cuprea* suggest that decoration disrupts solenoidal eddies as current moves past the tube, thereby reducing sediment scour (B. Little and M. LaBarbera, unpublished data). Crypsis has also been suggested, but experimental tests have not supported this hypothesis for *D. cuprea* [40]. Naturally, species such as *D. neapolitana* with non-emergent/non-decorated tubes would not be able to feed on the tube cap or enjoy benefits such as scour reduction; this raises questions about how *D. neapolitana* feeding and environmental tolerances differ from those of other members of the genus and how its ecosystem effects differ from those established for *D. cuprea* and others with emergent tubes. *D. neapolitana* appears to occupy lower-energy, muddier habitats than other species (S. Woodin, personal communication), perhaps rendering sediment scour less problematic. It is also sluggish and easier to dig in comparison to *D. cuprea* (personal observation) and *D. biscayensis* (S. Woodin, personal communication). This might point to differences in *D. neapolitana* metabolism and diet. Understanding how tube-worm–sediment interactions vary across the range of tube phenotypes in the genus would be an interesting area for further study.

*Diopatra* occupies physically dynamic habitats, subject to both erosion and deposition resulting from variable currents and storms. *D. cuprea* responds to erosion by trimming off excess tube material with the jaws, thus maintaining a tube cap with a height of roughly 2–5 cm. In response to sediment deposition, the worm extends the tube upward through the sediment and rapidly establishes a new tube cap [34]. This response to deposition and erosion has led some researchers to suggest that *Diopatra* tubes can be used to measure sediment dynamics at a given site [22,33]. Tube caps can be lost during storms or due to the activities of epibenthic organisms. It is common to see *Diopatra* caps in the wrack zone or accumulated in the bottom of stingray feeding pits (pers obs). During *Mogula* settlement season, worms may cut off tube caps that have become too overgrown with tunicates (S. Woodin, personal communication). *D. cuprea* can entirely rebuild a lost tube cap within ~12 hrs. For populations at high densities, *D. cuprea* tends to orient tubes either perpendicular or parallel to neighboring tubes (presumably allowing the worm to easily feed from its neighbor). At lower population densities, however, tubes are oriented perpendicular to the direction of prevailing currents. This may facilitate passive diffusion from the tube and/or could reduce sediment deposition inside the tube, particularly in oscillatory flow conditions [41]. In very high flows, the tube opening actually collapses, which may further prevent sediment influx [42].

## 3. Physical Effects of the Tube

Aggregations of polychaete tubes generally stabilize sediment, as evidenced by reduced ripple formation and the development of a diatom layer on the sediment surface within polychaete beds [43,44,45,46]. This is somewhat counterintuitive, given that eddies generated as water flows past tube structures cause upstream sediment scour and downstream deposition [47,48]. However, such destabilizing effects are evidently countered by multiple mechanisms that enhance stability. These include increased sediment binding due to mucous and microbial growth associated with animal activities [45,47,49,50], as well as skimming flow over tube beds [45,51]. Whereas specific conditions at any given site depend on local flow regimes, tube sizes, and tube density, tube mimics can create skimming flow when occupying as little as 8.8% of the available area [51]. This threshold would be met or exceeded in many *Diopatra* beds according to published field abundances (e.g., [31,52]), particularly when considering that tube decoration extends roughness well beyond the area occupied by the tube itself. Sediment deposition from skimming flow could well account for “mounds” associated with *D. leuckarti* and *D. ornata* in the Pacific Ocean [29,37]. 

It is possible that the tube helps *Diopatra* colonize mobile sediments, and their stabilizing effect makes the area suitable for other infauna; this hypothesis is supported by observations that a *Diopatra* species (referred to as *D. neapolitana* but almost certainly something else) was among the first species to colonize an eroded sand flat in Malaysia following disruption by a typhoon. Their establishment preceded colonization by other organisms [53].

## 4. Facilitation of Macroalgae and Plants

*Diopatra* interactions with macroalgae have been best established for *D. cuprea* in the northwestern Atlantic. By actively attaching drift algae to its tube, *D. cuprea* facilitates an algal canopy in habitats that would otherwise lack stable, attached algal populations (Table 1); *Diopatra* thus acts as a foundation species in a facilitation cascade [54,55]. Seagrasses, may also be facilitated when *D. cuprea* attaches reproductive shoots [56]. A single *D. cuprea* tube can support more than 300 mg of algal biomass representing multiple species [11]. Whereas the quantitative data for *Diopatra* facilitation of algae is exclusively from *D. cuprea,* one would expect similar effects for any species that builds similarly decorated tubes. It is worth noting, however, that even for *D. cuprea*, the interaction with algae can vary spatially. In habits with fast current, tubes constructed entirely of shell debris are often observed (personal observation), perhaps because drift algae passes by too quickly for worms to catch it and/or because algae would increase drag forces, leading to cap breakage in high flows. There also exists a well-documented latitudinal gradient in which *D. cuprea* decoration decreases dramatically in the southern portion of its range. This is driven by behavioral variability rather than by algal availability [11]. Whereas geographic patterns in behavior could point to a cryptic species complex, CO1 genetic patterns do not entirely mirror the behavioral patterns (Sotka et al., this issue). The mechanism underlying the behavioral variability remains an open question.

*D. cuprea*’s decorating behavior has notably been implicated in facilitating the invasive *Agarophyton vermiculophyllum* (formerly *Gracilaria vermiculophylla*) in the western Atlantic [52,57,58]. The extent to which *D. cuprea* has accelerated the invasion is difficult to quantify; *A. vermiculophyllum* is an aggressive invader in its own right and would undoubtedly have invaded even without *D. cuprea* (as is happening in many habitats worldwide [59]). However, *D. cuprea* indisputably anchors and stabilizes extensive *A. vermiculophyllum* mats. *A. vermiculophyllum* itself provides habitat for epibenthic invertebrates [58] and nursery habitat for juvenile blue crabs [60]. The *D. cuprea*–*A. vermiculophyllum* relationship may therefore be amplifying a habitat cascade, indirectly enhancing secondary productivity in some habitats [61]. However, two important caveats must be made: first, we do not know the extent to which *D. cuprea* + *A. vermiculophyllum* functions differently than *D. cuprea* + native macroalgae. Second, superblooms of *A. vermiculophyllum* have been associated with sediment anoxia and *D. cuprea* death [52], suggesting that facilitation of invertebrate communities only occurs below a threshold of *A. vermiculophyllum* abundance. As the invasion proceeds, it is unclear whether systems will stabilize in an enhanced-functioning state or an anoxic, reduced-functioning state.

**Table 1:** Studies quantifying *Diopatra* facilitation of algae and plants.

Study	Species	Location	Effects
Mangum et al., 1968 [31]	*D. cuprea*	Chesapeake Bay, Virginia, USA	20 algal species identified from tubes
Harwell and Orth 2001 [56]	*D. cuprea*	Chesapeake Bay, Virginia, USA	Tubes facilitate reproductive seagrass shoots
Thomsen 2004 [62]	*D. cuprea*	Hog Island Bay, Virginia, USA	Tubes facilitate *Ulva* and *A. vermiculophyllum*
Thomsen & McGlathery 2005; Thomsen et al., 2005 [57,63]	*D. cuprea*	Hog Island Bay, Virginia, USA	Tubes facilitate invasive alga *Agarophyton vermiculophyllum*
Berke 2012 [11]	*D. cuprea*	Northwest Atlantic (Massachusetts through Florida, USA)	Tubes support a total of 34 species (as many as 15 species within a single region)

## 5. Facilitation of Infauna

*Diopatra* tubes provide refugia for infauna by physically excluding predators, such as crabs, *Limulus*, epibenthic fish, skates, rays, and shorebirds (Table 2). This effect appears to be driven by the physical structure of the tube—tube mimics built of soda straws have the same effect [12]. In addition to predator exclusion, tube effects on local flow dynamics may promote entrainment of passively dispersing larvae, increasing recruitment near tubes [64]. Whereas most of the work on *Diopatra* facilitation of infauna has focused on *D. cuprea*, similar effects have been described for *D. ornata*, *D. leuckarti*, and others (Table 2).

Whereas *Diopatra* facilitation of infauna appears to be widespread, multiple factors can influence the strength of the interaction. For example, seasonal factors may contribute to variability; Santos and Aviz (2018) found that infauna on a beach with scattered *Diopatra* (densities of 25–75 m^−2^) were more diverse and abundant during the rainy season in comparison to a nearby site with no *Diopatra*. However, this effect was not observed during the dry season. Tube density can also be important; for example, Woodin [4,12] found that a density of 6 · 0.01 m^−2^ had a clear effect at an inlet site in Virignia, USA, whereas single tubes had no effect. Similarly, Bell and Woodin [65] tested zero, one, three, and six tubes in 0.01 m^−2^, finding significant differences for only the most extreme zero vs. six tube comparison. This is also consistent with Ban and Nelson’s [66] finding that four tubes in 0.01 m^−2^ had no effect at a subtropical site in the Indian River Lagoon, FL, USA. In contrast, Thomsen et al. (2011) found an effect of single onuphid tubes (presumed to be a *Diopatra* species) on a sand flat in Mozambique [23], whereas Santos and Avis (2018) found elevated density/abundance in an area with scattered single *Diopatra* tubes in Brazil [20] (referred to as *D. cuprea*, although *Diopatra* taxonomy in this region has been recently revised). Both of these studies make it somewhat difficult to disambiguate effects of the tube-cap fauna from true infauna; Thomsen et al. [23] compared cores with sediment + tube cap to tube caps alone, making it possible to infer that infauna were affected separately from the tube-cap fauna. In the Santos and Avis study [20], infauna and the tube-cap fauna were analyzed together, so disambiguation is not possible. Some variability in *Diopatra* effects on infauna might reflect differences in local hydrodynamic regimes and subsequent effects on larval recruitment. Variability could also stem from geographic and seasonal differences in processes such as recruitment and predator abundance. If tubes are physically excluding predators, then one would expect predator size distributions and specific foraging behaviors to play a role. Interactions among smaller infauna may also be important; for example, when facilitated infauna prey on smaller meiofauna and juvenile bivalves, complex patterns can emerge [65,67].

Interestingly, even as *Diopatra* facilitates other infauna, the worm itself might be sensitive to competition, particularly at the settlement stage. Flamingo exclusion structures in Namibia reduced *Diopatra* abundance, even as all other infauna increased [68] (referred to as *D. neapolitana*, although Namibia is far outside its range). This contrasts with Woodin’s (1981) finding of enhanced *D. cuprea* recruitment in predator-exclusion cages in Virginia, USA. This contrast emphasizes that we have much to learn about the processes influencing *Diopatra* recruitment in different systems.

**Table 2:** Studies quantifying *Diopatra* facilitation of infauna.

Study	Species	Location	Densities Tested	Effects
Woodin 1978 [4]	*D. cuprea*	Tom’s Cove, Virginia, USA	0, 1, 6 · 0.01 m^−2^	↑ infaunal richness and abundance at 6 · 0.01 m^−2^
Woodin 1981 [12]	*D. cuprea*	Tom’s Cove, Virginia, USA	0, 1, 6 · 0.01 m^−2^	↑ infaunal abundance at 6 · 0.01 m^−2^
Bell and Woodin 1984 [65]	*D. cuprea*	Tom’s Cove, Virginia, USA	0, 1, 3, 6 · 0.01 m^−2^	↑ polychaete abundance at 6 · 0.01 m^−2^; no effect on meiofauna
Bailey-Brock 1984 [29]	*D. leuckarti*	Niu Valley, Hawaii, USA	“mounds” up to 21,800 m^−2^	28 species from 7 phyla are associated with mounds
Luckenbach 1984 [69]	*D. cuprea*	North Inlet, South Carolina, USA	0, ≥9 · 0.01 m^−2^	↑ infaunal abundance near tubes
Luckenbach 1984 [70]	*D. cuprea*	North Inlet, South Carolina, USA	0, ≥10 · 0.01 m^−2^	↑ infaunal abundance in areas of high tube density
Ban and Nelson 1987 [66]	*D. cuprea*	Indian River Lagoon, Florida, USA	0, 1, 4 · 0.01 m^−2^	No effect
Ambrose & Anderson 1990 [71]	*D. ornata*	Pendleton Artificial Reef, California, USA	Inside vs. outside “beds”	↑ richness and abundance of infauna and decapods
Thomsen et al., 2011 [23]	*Diopatra-*like onuphid *	Inhaca Island, Mozambique	0, 1 · 0.01 m^−2^	↑ richness and abundance around single tubes
Santos and Aviz 2018 [20] ^†^	*D. cuprea* *	Algodoal-Maiandeua, Brazil	0 vs. 25–75 m^−2^, but each sample from the *Diopatra* area included only 1	Seasonal ↑ richness and abundance

* Species in this region have been recently revised. ^†^ Sampling captured both infauna and the tube-cap, making it impossible to separate infauna from epifauna.

## 6. Facilitation of Epibenthic Fauna

*Diopatra* tubes directly provide habitat for smaller epibenthic fauna, including a diverse array of protists, meiofauna, gastropods, bivalves, amphipods, cnidarians, flatworms, bryozoans, and tunicates (Table 3). Tube-cap fauna are most strongly facilitated when tubes are decorated with macroalgae, although caps lacking algae do also support an epifaunal community [58,72,73]. Larger organisms, such as juvenile fish and crabs, also utilize *Diopatra* tubes. In the northwestern Atlantic, juvenile fish show a diel pattern of habitat use, spending daylight hours sheltering in *D. cuprea* beds but venturing into open habitats to forage at night [10]. Mounds of *D. leuckarti* and *D. ornata* support elevated diversity and abundance of epibenthic crabs and shrimp [29,71]. In habitats where *Diopatra* facilitates *A. vermiculophyllum* (e.g., most of the US Atlantic coast), we would also expect it to indirectly facilitate juvenile blue crab, which have higher survivorship in *A. vermiculophyllum* [55,60].

## 7. Food Web Connections

*Diopatra* are omnivores, primarily feeding on organisms living on the tube cap, on neighboring tube caps, and within nearby sediments. *D. cuprea* is capable of extending 10 cm or more from the tube but appears to spend most of its time grazing within a radius <5 cm (pers. obs.). This species exhibits a strong feeding response to extracts of other polychaetes, bivalves, and *Artemia* but also shows a weaker feeding response to other arthropods, echinoderms, *Fundulus, Codium*, and *Zostera* [75]. Gut contents include a diverse array of invertebrates, protists, *Zostera*, chlorophytes, rhodophytes, and phaeophytes [31], and I have seen worms eat *Ulva* in the laboratory. 

*Diopatra* will scavenge to at least some extent; in North Inlet SC, I once encountered a number of dead minnows littering a *D. cuprea* flat. At least one worm had attached a fish to its tube cap, and another doggedly attempted to pull a fish into its tube, seemingly undaunted by the physical impossibility of this task. That said, *D. cuprea* held in the lab will ignore shrimp meat that is more than a day old, so true scavenging seems unlikely.

*Diopatra* are found in the guts of multiple epibenthic fish, including flounder, skates, pigfish, croaker, and turbot [76,77,78]. One report also describes the large gastropod *Fasciolaria hunteria* feeding on *D. cuprea* (and the similar *Americonuphis magna*) by inserting its long proboscis down the tube and rasping at the worm’s tissue [79]. *Diopatra* avoids predation by withdrawing rapidly to depth; the strength of this response varies within the genus, as indicated by the difficulty of collecting worms. For example, *D. cuprea* requires skill to dig; the worm is quite vibration-sensitive, so one must step gingerly and strike rapidly with the shovel. In contrast, I was surprised by how easy it was to dig *D. neapolitana* in Europe. I suspect this difference is at least partly why *D. neapolitana* is widely harvested for fishing bait [28,80,81], whereas *D. cuprea* is not. 

Because *Diopatra* can regenerate both anteriorly and posteriorly, predation attempts are not always lethal ([81] and references therein). The worm can cling very tightly to the tube, using hydrostatic pressure to press the chaetae into the textured tube wall [82]. This, together with the tube’s depth, make it generally impossible to pull a worm entirely out of its tube, as the worm will simply break in the attempt. Unsurprisingly, then, worms undergoing anterior regeneration are commonly collected in the field. These represent anywhere from 5–40% of specimens, depending on the species, place, and season (summarized in [81]). Antennae are also frequently nipped off by predators, and antennal regeneration is even more common than head regeneration [83]. 

## 8. Human Impacts

*Diopatra* species are impacted by human activities on multiple fronts; climate change, harvesting for the bait trade, human-assisted transport, and interactions with invasive species are all important aspects of *Diopatra* biogeography and ecology. 

### 8.1. Range Expansions and Climate Change

*Diopatra* occupies tropical and temperate waters, with poleward range limits evidently set by cold temperature limits on summer reproduction; *D. neapolitana* and *D. biscayensis* do not occur at sites where August sea-surface temperature (SST) remains below 18 °C, suggesting that warmer temperatures are needed for successful reproduction [36,84]. Cold winter temperatures may also play a role by inhibiting feeding and tube maintenance [34,75,85]. At cold temperatures, *D. cuprea* stops feeding between 5–8 °C [75,85] and stops maintaining its tube below 1.8 °C [34]. We would therefore expect *Diopatra* ranges to extend poleward with climate change, as has been demonstrated for *D. biscayensis* in western Europe, where the worm’s progressive northward expansion has matched warm SST anomalies in the Bay of Biscay [84]. The *D. biscayensis* range shift has been accelerated by human transport, most likely associated with mussel aquaculture [36,86,87].

In the northwestern Atlantic, we have not seen similar poleward movement in *D. cuprea* [35]—at least, not as of 2022 (Berke unpublished data). This is somewhat surprising, as the Gulf of Maine has been warming rapidly [88]. Whether this reflects different thermal biology, constraints of larval supply, lack of intertidal aquacultural transport, or something else remains an open question. In Europe, the lecithotrophic larvae of *D. neapolitana* and *D. biscayensis* both have a short pelagic period that limits their dispersal ability; range shifts in *D. biscayensis* are therefore human-assisted [86,87]. *D. cuprea* appears to have a similarly short larval period [89], which may account for its as-yet stable northern range limit.

The extent to which *Diopatra* will be impacted by higher temperatures in tropical and subtropical habitats remains largely unexplored. *Diopatra* thermal tolerances vary seasonally and geographically; a 1969 study found that temperatures of 37–38 °C were 100% lethal for *D. cuprea* from Barnstable, Massachusetts, and 50% lethal for worms from Beaufort, NC, during winter months. However, Beaufort worms acclimatized in summer months to a 50% lethal limit near 42 °C. Linking such laboratory data to field conditions will be complicated by the need to understand what temperatures worms actually experience in situ. Benthic temperatures can depart substantially from SSTs, and intertidal organisms can experience temperatures considerably different from the surrounding habitat [90,91,92,93,94]. Whereas models have been developed to estimate sediment surface temperature for intertidal mud flats [95], infauna will experience progressively lower temperatures as their burrowing depth increases [96]. *Diopatra’s* tube extends much deeper into the sediment than most infauna, which should buffer it against surface extremes. Furthermore, worms must irrigate the tubes with overlying water and must partly emerge from the tube to forage, so exposure to ambient water temperatures could be substantial. Understanding how worms such as *Diopatra* experience temperature over the course of a day, season, or year and how this would differ for intertidal vs. subtidal populations are interesting questions. For the lugworm *Arenicola marina,* temperatures at burrow depths appear to correlate well with SST, allowing biogeographic models to predict likely responses to warming oceans [94]. Whereas lugworm burrows are shallower and more permeable than *Diopatra* tubes, this modeling approach holds considerable promise for understanding climate responses in *Diopatra* and infaunal organisms in general. 

### 8.2. Bait Harvesting

Throughout western Europe, intertidal *Diopatra* are dug for use as fishing bait. In Portugal’s Canal de Mira (Ria de Aveiro estuary), as many as 4.3 million individual *D. neapolitana* may be harvested each year [28,97,98], with an economic value of more than EUR 325,000/yr [28]. Whereas *D. neapolitana* can regenerate anterior segments, this ability is limited to the first 15–20 chaetigers, and bait diggers typically collect more than that, suggesting that the activity is generally lethal [81]. Modeling based on catch per unit effort has suggested that *D. neapolitana* harvesting remains short of maximum sustainable yield for the system [98]. However, Cunha et al. [28] estimated that diggers collected roughly 2.9 worms · m^−2^ over a period of a few winter days, a number that is alarmingly close to their estimate for the standing population density of 2.8 worms · m^−2^. Both estimates were based on a fairly limited one-time field survey, so we should not derive too many conclusions (as the authors themselves emphasize). Nonetheless, the disconnect between this field survey and CPUE models highlights the need for a much more complete understanding of *D. neapolitana* population dynamics and the long-term sustainability of harvesting pressure on this species.

### 8.3. Interactions with Invasive Species

Given that *Diopatra* facilitate both algae and invertebrates, the potential for facilitating invasions is high. In the western Atlantic, *D. cuprea* is facilitating the invasive red alga *Agarophyton vermiculophyllum* [52,57,59,99]. *A. vermiculophyllum* is an aggressive invader in its own right and would likely be spreading even without *D. cuprea* facilitation; it indisputably forms thick mats anchored to *D. cuprea* tubes. These mats can provide habitat for smaller invertebrates [58,60] which, in turn, increases *D. cuprea* foraging opportunities [100], so effects of this invader are not universally negative. However, *A. vermiculophyllum* mats can also create anoxia in the sediment, which has been associated with mass *D. cuprea* die-offs, possibly contributing to long-term population declines throughout the mid-Atlantic US [52]. Conditions leading to *A. vermiculophyllum* blooms, their frequency, and effects on invertebrates, including *D. cuprea*, remain open questions. 

Whereas *Diopatra* species are not generally invasive, the population of *D. biscayensis* occurring north of the Brittany peninsula in France is evidently the result of human transport from the Bay of Biscay [86,87]. This raises the possibility that other *Diopatra* species could possibly have been moved through aquaculture and the bait trade. Most *Diopatra* appear to have a short-lived larval period, making transport in ballast water unlikely, but juveniles could be transported as hitch-hikers in mud and algae associated with aquaculture [87]. As *D. biscayensis* expands north, it will introduce large tube structures into areas previously dominated by the bioturbator *A. marina*. This change would be expected to result in an overall increase in sediment stability, productivity, and local diversity [84]. As new *Diopatra* species are described, researchers should bear in mind the possibility of human-assisted transport.

## 9. Future Directions

*Diopatra* occur worldwide and are ecologically important members of coastal sedimentary communities. However, in many ways, we know more about the worm’s effects on other organisms than we know about the worm itself; surprisingly little has been published about *Diopatra* autecology and basic biology. We generally need better understanding of the worm’s environmental tolerances, feeding biology, reproductive cycles, and population dynamics for all *Diopatra* species, especially in populations subject to harvesting or existing near geographic range limits. In particular, understanding thermal biology and responses to climate change will require learning more about the relationship between SST, bottom temperatures, and an individual *Diopatra*’s experienced temperature.

These gaps in our knowledge become especially evident as new species are identified. Taxonomists have made enormous progress in recent years, disentangling species complexes and revealing hitherto unknown diversity. Comparing and contrasting the ecology and ecosystem engineering effects of different *Diopatra* in different localities should be a priority. This work will not be possible without close collaboration between ecologists and systematists. Funding to support taxonomic work alongside ecological investigations is critically important. Understanding how ecosystem engineering effects vary with *Diopatra* species, habitat, and season may help us better understand how predation, competition, recruitment, and facilitation structure sedimentary communities writ large.

## Figures and Tables

**Table 3:** Studies quantifying *Diopatra* facilitation of epibenthic fauna.

Study	Species	Location	Community Type	Densities Tested	Effects
Mangum et al., 1968 [31]	*D. cuprea*	Chesapeake Bay, Virginia, USA	Epifauna	Single tubes	Tubes support 49 species of Arthropoda, Mollusca, Annelida, and other invertebrate phyla.
Bell & Coen 1982 [9]	*D. cuprea*	Tampa Bay, FL	Meiofauna	Single tubes	Tubes support crustacean nauplii, copepods, and nematodes (tens to hundreds per tube). Polychaetes also found on tubes.
Bell & Coen 1982 [72]	*D. cuprea*	Tom’s Cove, Virginia, USA	Meiofauna	1 or 4 · 0.01 m^−2^	Tubes support nauplii, copepods, amphipods, ostracods, nematodes, and polychaetes. Abundances increase when algae are also present, with no effect of tube density.
Dudley et al., 1989 [74]	*D. ornata*	Venado Beach, Panama	Epifaunal mollusca	Single tubes	Tubes support seven species of gastropod, four bivalves, and a chiton.
Dudley et al., 1989 [74]	*D. cuprea*	Virginia, USA and Woods Hole, Massachusetts, USA	Epifaunal mollusca	Single tubes	Tubes support five species of gastropod and four bivalves.
Diaz et al., 2003 [10]	*D. cuprea*	Mid-Atlantic Bight	Juvenile epibenthic fish	“mat”	Juvenile fish were twice as abundant in tube mats as bare sand during the day (reversed at night). Eight species of fish were associated with D. cuprea tubes.
Thomsen et al., 2011 [23]	*Diopatra-*like onuphid *	Inhaca Island, Mozambique	Epifauna	Single tubes	↑ richness and abundance on single tubes
Santos and Aviz 2018 [20] ^†^	*D. cuprea* *	Algodoal-Maiandeua, Brazil	Epifauna and Infauna	Single tubes	Seasonal ↑ richness and abundance

* Species in this region have been recently revised, ^†^ Sampling captured both infauna and the tube-cap, making it impossible to separate infauna from epifauna.
